# Modulation-Based Feature Extraction for Robust Sleep Stage Classification Across Apnea-Based Cohorts

**DOI:** 10.3390/bios16010056

**Published:** 2026-01-13

**Authors:** Unaza Tallal, Rupesh Agrawal, Shruti Kshirsagar

**Affiliations:** 1 School of Computing, Wichita State University, 1845 N, Wichita, KS 67208, USA; shruti.kshirsagar@wichita.edu; 2 Business Informatics Department, Northern Kentucky University, Highland Heights, KY 41099, USA; agrawalr1@nku.edu

**Keywords:** automated sleep staging, obstructive sleep apnea, modulation spectrogram, time–frequency analysis, EEG signal processing

## Abstract

Automated sleep staging remains challenging due to the transitional nature of certain sleep stages, particularly N1. In this paper, we explore modulation spectrograms for automatic sleep staging to capture the transitional nature of sleep stages and compare them with conventional benchmark features, such as the Short-Time Fourier Transform (STFT) and the Continuous Wavelet Transform (CWT). We utilized a single-channel EEG (C4–M1) from the DREAMT dataset with subject-independent validation. We stratify participants by the Apnea–Hypopnea Index (AHI) into Normal, Mild, Moderate, and Severe groups to assess clinical generalizability. Our modulation-based framework significantly outperforms STFT and CWT in the Mild and Severe cohorts, while maintaining comparable high performance in the Normal and Moderate AHI groups. Notably, the proposed framework maintained robust performance in severe apnea cohorts, effectively mitigating the degradation observed in standard time–frequency baselines. These findings demonstrate the effectiveness of modulation spectrograms for sleep staging while emphasizing the importance of medical stratification for reliable outcomes in clinical populations.

## 1. Introduction

Sleep is a physiological human need that influences cognitive performance, physical health, and mental well-being. With industrialization and human anthropological changes, sleep disorders are becoming increasingly prevalent worldwide [1]. Growth in remote health monitoring and diagnostic devices has led to clinical support for their use [2]. With the exponential growth and adoption of artificial intelligence (AI), the need for more effective diagnostics is driving the exploration of new AI approaches and feature-extraction methodologies. The rapid growth of technologies and the need for remote monitoring are driving innovation to improve the understanding of sleep architecture for overall human health.

Currently, polysomnography (PSG) remains the gold standard for diagnosing sleep health and quality. PSG is a lab-based sleep study diagnostic test that involves continuous monitoring of physiological signals, such as the Encephalogram (EEG), electrocardiogram (ECG), electrooculogram (EOG), respiratory signals, snoring, and oxygen saturation (SpO_2_) [3]. Among these modalities, EEG offers an efficient means of capturing brain activity because it is non-invasive, inexpensive compared to other neuroimaging modalities, and provides superior temporal resolution. Different EEG frequency bands, including delta (0.5–4 Hz), theta (4–8 Hz), alpha (8–12 Hz), sigma (12–15 Hz), and beta (15–30 Hz), are closely associated with specific sleep stages and thus commonly serve as features for sleep stage classification [4].

Despite the richness of EEG frequency bands and recent advances in deep learning methods for sleep stage classification, certain transitional stages, particularly N1 or light sleep stage, remain challenging to predict with high accuracy due to overlapping spectral patterns between wake and light-sleep phases, as well as their brief and ambiguous characteristics [5]. These limitations have drawn researchers’ attention to explore robust feature-extraction techniques that can capture both the spectral content and subtle temporal physiological signal patterns. Time–frequency and spectral analyses have shown potential in extracting distinctive patterns related to sleep-specific characteristics [6]; however, more efficient feature-extraction methods are needed to encode fine-grained frequency components and characterize slow temporal modulations to capture sleep dynamics.

Traditional spectrotemporal methods, such as the Short-Time Fourier Transform (STFT), precisely map spectral variations across time [7]; however, they cannot explicitly model the rate of amplitude modulations within frequency bands. Recent advancements have attempted to capture these temporal dynamics through architectural innovations, including multi-scale approaches [8], temporal attention mechanisms [9], and envelope-based feature extraction [10]. Yet, these methods fundamentally operate on standard inputs; the quality of the initial representation strictly bounds their performance. In contrast, Modulation analysis that can decompose signals into carrier and modulation frequencies [11], introduces a modulation axis missing from time–frequency inputs. By representing both the spectral content of brain rhythms and their time-varying amplitude envelopes, modulation spectrograms can capture discriminative features better than conventional time–frequency methods [12]. The existing literature indicates that modulation spectrograms remain unexplored for comprehensive sleep classification across all five (5) canonical stages (Awake, N1, N2, N3, REM). The current study leverages modulation spectrogram features within a deep neural network framework for 5-class sleep-stage classification.

Despite advances in sleep staging algorithms, most methods are developed and validated on predominantly healthy cohorts or mixed populations without stratification by clinical severity [8,13,14,15]. However, obstructive sleep apnea (OSA), which affects millions globally, fundamentally disrupts sleep architecture through repeated breathing interruptions, resulting in increased sleep fragmentation, stage instability, and abnormal EEG patterns [16,17,18]. The Apnea–Hypopnea Index (AHI), which quantifies respiratory event frequency, directly correlates with the degree of sleep disruption [19,20,21]: patients with severe OSA (AHI ≥ 30) experience markedly different sleep dynamics than those with moderate (AHI 15–29), mild OSA (AHI 5–14) or normal sleep (AHI < 5). Consequently, algorithms trained on mixed populations may exhibit unpredictable performance degradation in clinical settings where OSA is prevalent [22]. To ensure clinical utility and generalizability, sleep staging methods must demonstrate robust performance across AHI-stratified groups, yet few studies systematically evaluate this critical dimension [23,24]. We address this gap by validating our modulation-based approach across Normal, Mild, Moderate, and Severe AHI cohorts, providing evidence of robustness in populations most likely to require automated sleep staging in clinical practice.

The main contributions of this work are as follows:

1. We introduce a domain adaptive modulation spectrograms framework that works beyond the standard ‘out-of-the-box’ application of time–frequency representations. This framework employs a novel constraint-aware windowing mechanism that reconciles the trade-off between the high-frequency resolution required for modulation features (60 s context) and the strict temporal localization required for clinical scoring (30 s epoch). By effectively integrating these constraints, the proposed method captures both spectral content and amplitude-modulation patterns that characterize sleep architecture, demonstrating particular effectiveness in identifying challenging transitional stages such as N1.
2. We systematically demonstrate that our proposed methodology offers superior robustness against sleep fragmentation compared to the baseline. By evaluating Apnea–Hypopnea Index (AHI)-stratified cohorts (Normal, Mild, Moderate, and Severe), we show that as sleep apnea severity increases, the baseline performance degrades significantly. The proposed framework demonstrates stable performance in patient populations, addressing a critical gap in clinical generalizability.
3. We conduct detailed ablation studies to identify optimal configurations for modulation-based features, including window length and image resolution, providing evidence-based guidelines for implementation and establishing a more generalizable and efficient method.

The rest of this paper is organized as follows: Section 2 reviews related work on sleep stage classification and feature extraction methods. Section 3 describes the proposed methods, including the dataset and AHI-based stratification and modulation spectrogram framework. Section 4 details the experimental setup, including the data preprocessing, deep learning architecture, training protocol, evaluation metrics, and benchmark systems. Section 5 presents results and discusses our findings, including ablation study results, baseline comparisons, and clinical implications, limitations, and outlines future research directions. Finally, Section 6 provides the conclusion of the study.

## 2. Related Work

In this section, we review related work on feature extraction methods for automated sleep staging, deep learning architectures for classification, and the clinical validation of sleep staging systems across patient populations with varying obstructive sleep apnea severity.

(A) Feature representation: Automatic sleep stage classification fundamentally relies on extracting discriminative features from physiological signals [13,25]. Early systems used hand-crafted features derived from expert knowledge, including power spectral density in standard EEG frequency bands (delta, theta, alpha, sigma, beta), statistical measures, and specific event detections such as sleep spindles and K-complexes [4]. While these features encoded valuable domain knowledge, they required extensive manual engineering. They could miss discriminative patterns not anticipated by human experts, motivating researchers to explore methods that could automatically learn optimal feature representations from data [8]. Time–frequency analysis emerged as a powerful approach for sleep-feature extraction because sleep EEG is fundamentally non-stationary, its frequency content changes as sleep stages evolve [26]. The Short-Time Fourier Transform (STFT) has become widely adopted for generating spectrograms that visualize how spectral content evolves [7]. However, the Short-Time Fourier Transform (STFT) suffers from a fixed time–frequency resolution trade-off: short windows provide good temporal localization but poor frequency resolution, while long windows provide the opposite [27]. To address this limitation, Continuous Wavelet Transform (CWT) provides adaptive multi-resolution analysis, yielding fine frequency resolution at low frequencies for slow oscillations and fine temporal resolution at high frequencies for rapid transients like sleep spindles [28]. Comparative studies have systematically evaluated different feature extraction methods to identify optimal representations. Ref. [29] compared multiple transformation methods, including CWT, STFT, FFT-based features, and recurrence plots using identical deep learning architectures, demonstrating that feature representation choice significantly impacts performance, often more than architectural innovations. Similarly, Ref. [30] found that time–frequency representations consistently outperformed raw signals or simple spectral features across different network architectures. These studies establish that feature representation is critical for effective sleep staging, with time–frequency methods generally providing superior performance by capturing both spectral content and temporal dynamics. Despite extensive exploration of time–frequency methods, existing approaches share a common limitation: they focus on instantaneous frequency content but do not explicitly model amplitude modulation patterns that characterize many sleep phenomena [31,32]. N2 sleep spindles exhibit characteristic waxing-waning amplitude envelopes, K-complexes show sharp amplitude transients, and N3 slow-wave activity displays distinctive amplitude fluctuation patterns. While STFT and CWT detect oscillations at specific frequencies and times, they do not explicitly represent how amplitude varies over time [31]. Modulation analysis addresses this gap by decomposing signals into carrier frequencies (spectral content) and modulation frequencies (rate of amplitude variation), producing modulation spectrograms that explicitly represent both dimensions [11]. Although modulation features have proven valuable in speech processing [33] and have shown promise in biomedical applications [12], they remain largely unexplored for sleep staging despite the prevalence of amplitude-modulated phenomena in sleep EEG.
(B) Classification methods: For classification, deep learning has replaced traditional machine learning methods due to its ability to learn hierarchical representations. Convolutional neural networks (CNNs) have become the dominant approach for processing time–frequency representations of EEG signals, with numerous studies demonstrating their effectiveness on STFT spectrograms [34,35,36]. These architectures automatically learn spatial filters that detect discriminative patterns in spectrograms, such as sleep spindles, K-complexes, and characteristic frequency band signatures across different sleep stages [37]. Recurrent neural networks model temporal dependencies across consecutive sleep epochs. Ref. [8] introduced DeepSleepNet, combining CNNs with bidirectional LSTM to capture temporal context. More recently, attention mechanisms [9] and transformer architectures [38] have been explored to capture long-range dependencies. However, these require larger datasets and may not provide proportional gains when using well-designed feature representations. Ref. [39] proposed EEGSNet, integrating residual CNN blocks, bidirectional LSTM, and an auxiliary classifier for intermediate supervision, demonstrating strong performance across multiple datasets. We adopt EEGSNet as our backbone architecture to isolate the contribution of modulation-based features by maintaining a consistent classification framework across all feature types.
(C) Clinical validation across patient populations: While significant progress has been made in developing sophisticated feature extraction methods and deep learning architectures for sleep staging, the systematic evaluation of these systems across clinically diverse patient populations remains critically underexplored. The majority of benchmark sleep datasets used in algorithm development including Sleep-EDF [8], MASS [13], and Sleep-EDFX [30] consist primarily of healthy subjects or heterogeneous populations without stratification by clinical severity. This evaluation paradigm may overestimate real-world performance, as algorithms deployed in clinical settings predominantly encounter patients with sleep disorders whose physiological characteristics differ substantially from healthy cohorts.
  Obstructive sleep apnea (OSA), one of the most prevalent sleep disorders affecting an estimated 425 million adults globally [17], fundamentally alters sleep architecture in ways that challenge automated staging systems. OSA is characterized by repeated upper airway obstructions during sleep, leading to intermittent hypoxemia, recurrent arousals, and sleep fragmentation [16,20]. These respiratory events disrupt normal sleep stage progression, resulting in increased stage transitions, reduced slow-wave sleep, fragmented REM sleep, and typical EEG patterns that may confound classification algorithms trained on healthy populations [21]. The Apnea–Hypopnea Index (AHI), defined as the average number of apnea and hypopnea events per hour of sleep [19], provides a clinically validated measure of OSA severity: AHI < 5 indicates normal sleep, AHI 5–14 indicates mild OSA, AHI 15–29 indicates moderate OSA, and AHI ≥ 30 indicates severe OSA [20].
  Recent work has begun to address this validation gap. Ref. [40] introduced the DREAMT dataset specifically designed to assess sleep staging algorithms on a population with sleep disorders, demonstrating that model performance varies significantly across demographic subgroups and highlighting systematic biases in algorithms trained on predominantly healthy cohorts. Ref. [5] proposed a two-branch neural network architecture explicitly designed to balance performance across multiple cohorts with varying sleep disorder prevalence, recognizing that standard training procedures may optimize for majority patterns while underperforming on clinical subpopulations. However, despite these initial efforts, few studies systematically evaluate sleep staging performance across AHI-stratified severity groups, and most published work reports only aggregate metrics across mixed populations.
  The clinical implications of this validation gap are substantial. Sleep stage classification directly informs treatment decisions, including CPAP titration for OSA patients, surgical candidacy assessment, and monitoring of therapeutic response [19]. Algorithms that perform well on healthy subjects but degrade in OSA populations risk misclassifying sleep architecture precisely in patients who most need an accurate assessment. Furthermore, the relationship between AHI severity and algorithm performance remains poorly characterized: it is unclear whether performance degrades linearly with increasing apnea severity, whether specific sleep stages become more difficult to classify in OSA populations, or whether specific feature extraction methods exhibit greater robustness to OSA-related EEG alterations.
  This work addresses these gaps by systematically evaluating modulation-based feature extraction across AHI-stratified cohorts (Normal, Mild, Moderate, and Severe) using the DREAMT dataset [40]. By comparing performance across severity groups and analyzing per-stage classification metrics, we provide insight into both the clinical generalizability of modulation spectrograms and the specific challenges posed by OSA populations for automated sleep staging systems. This stratified validation approach represents a critical step toward developing sleep staging algorithms that maintain robust performance across the diverse clinical populations encountered in real-world deployment.

## 3. Proposed Method

**Figure 1:**
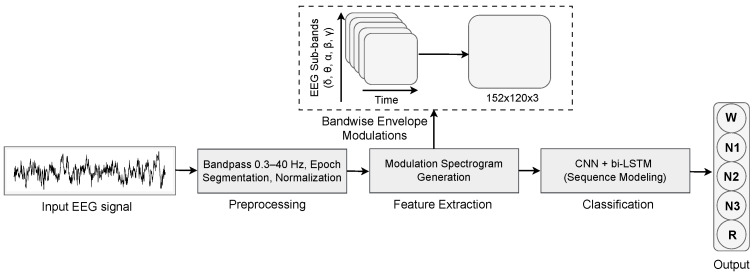
Block diagram of modulation-based sleep stage classification method.

This section describes the proposed pipeline for sleep stage classification based on the modulation spectrogram and an AHI-based cohort. Figure 1 depicts a block diagram illustrating the generation of a modulation spectrogram and subsequent classification of sleep stages. More details about each block are presented in the following sections.

### 3.1. Dataset and AHI-Based Stratification

In this study, we employ the DREAMT dataset (Dataset for Real-time sleep stage Estimation using Multi-sensor wearable Technology) [40] to evaluate the effectiveness of the proposed feature extraction method. This publicly available, open-source dataset includes overnight polysomnography (PSG) recordings alongside multi-sensor wearable recordings (Empatica E4) for each subject. Certified scorers annotated sleep stages in accordance with American Academy of Sleep Medicine (AASM) guidelines. This dataset has five sleep-stage categories: Wake, NREM (N1, N2, N3), and REM. The dataset comprises recordings from 100 participants recruited by the Duke Sleep Disorders Lab, including 45 males and 55 females aged 21 to 87 years (mean: 56.2 ± 16.6 years). Participants presented a range of health conditions, including hypertension, diabetes, obstructive sleep apnea, anxiety, and other sleep-related issues such as breathing difficulty and snoring. We stratified participants based on Apnea–Hypopnea Index (AHI) before model training to demonstrate that medical cohorts require different treatment than healthy cohorts for optimal results. Table 1 presents participant stratification and the number of epochs per class for each group.

### 3.2. Modulation Spectrogram

The modulation spectral signal representation has been described in detail in [33,41] and is summarized here for completeness.

**Figure 2:**
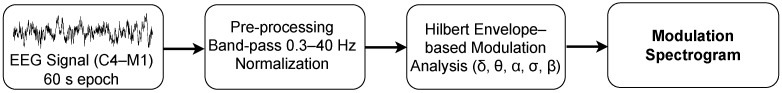
Workflow of modulation spectrogram generation.

The signal processing pipeline for computing modulation spectrograms from sleep EEG is illustrated in Figure 2. We maintain the sampling rate of channel C4–M1 at 100 Hz to preserve temporal resolution and apply a fourth-order Butterworth bandpass filter (0.3–40 Hz) to suppress low-frequency drifts and high-frequency noise while retaining the full range of sleep-related EEG oscillations. Each epoch undergoes z-score normalization to standardize amplitude variations across subjects and recordings. This step addresses inter-subject variability arising from differences in skull conductivity and electrode impedance. For a given subject *s*, the raw signal x(t) was standardized as:

$$x_{norm}\left(t\right)=\frac{x\left(t\right)-\mu_{s}}{\sigma_{s}}$$

where $\mu_{s}$ and $\sigma_{s}$ represent the global mean and standard deviation of the subject’s entire recording. This ensures that the subsequent feature extraction (Modulation Spectrogram) is invariant to absolute signal amplitude, focusing instead on relative spectral dynamics.

We implemented an adaptive windowing protocol to capture contextual information while maintaining strict alignment with clinical scoring definitions. For each target 30-s epoch $E_{i}$ with clinical label $y_{i}$, the input spectrogram is generated based on the stability of sleep stage relative to subsequent epoch $E_{i+1}$:

- Extended Window (60 s): If the sleep stage is stable ($y_{i}=y_{i+1}$), we utilize a window length of L=60 s covering the interval $\left[t_{i},t_{i+2}\right]$. This adheres to a strict agreement rule, ensuring the label $y_{i}$ represents the entire duration of the window.
- Fallback Window (30 s): If a transition occurs where ($y_{i}\neq y_{i+1}$), we restrict the window length to the standard L=30 s $\left[t_{i},t_{i+1}\right]$ to preserve the temporal boundry of transition.

This approach ensures complete data retention. In both cases, the label assigned to the resulting spectrogram is strictly $y_{i}$, eliminating the ambiguity of majority voting or center-epoch alignment. Following windowing, the preprocessed signal is decomposed into five physiologically meaningful subbands consistent with the AASM manual [19]: δ (0.5–4 Hz, slow-wave activity), θ (4–8 Hz, light sleep and drowsiness), α (8–12 Hz, relaxed wakefulness), σ (12–15 Hz, sleep spindles), and β (15–30 Hz, arousal and REM sleep). These bands capture the characteristic oscillatory signatures of different sleep stages [26]. We prioritized these fixed boundaries over data-driven or adaptive methods to maintain direct clinical interpretability and facilitate standard comparisons. For each subband, we extract the analytic envelope using the Hilbert transform, representing the instantaneous amplitude modulation of the carrier frequency. The envelopes are then low-pass filtered at 10 Hz and downsampled to 20 Hz to preserve modulation frequencies ≤10 Hz, which encompass the slow amplitude fluctuations characteristic of sleep phenomena such as waxing-waning sleep spindles and K-complex envelopes. This cutoff is physically motivated to capture the slow envelopes of cortical events (e.g., 1–2 Hz spindle oscillations) while functioning as a low-pass ‘smoothness constraint’ that rejects rapid fluctuations associated with EMG artifacts and non-biological noise [12,42]. The modulation spectrum is computed via the Fourier transform of each filtered envelope and normalized to the 6–99.6% range to enhance contrast.

The resulting modulation spectrogram is a two-dimensional representation where the horizontal axis encodes modulation frequency (rate of amplitude variation, 0–10 Hz) and the vertical axis encodes carrier frequency (spectral content, 0.5–40 Hz). This explicitly models both the spectral characteristics of brain rhythms and their temporal amplitude dynamics. We generate spectrograms at three image resolutions: 76 × 60 × 3 (baseline comparison), 152 × 120 × 3 (enhanced resolution), and 224 × 224 × 3 (standard CNN input).

## 4. Experimental Setup

In this section, we describe the data preprocessing, proposed model architectures, training protocol, evaluation metrics, and benchmark systems used to gauge system performance.

### 4.1. Data Preprocessing

We use the DREAMT dataset in this study, which we stratify using the Apnea–Hypopnea Index (AHI) as discussed in Section 3.1. We preprocessed the dataset and then applied the feature extraction method. Next, we perform subject-independent classification to ensure the generalizability of the EEGSNet architecture and to prevent data leakage within subjects. The dataset is carefully organized to avoid subject overlap by using longer temporal windows, and a held-out test set is reserved for final evaluation, while the validation set supports parameter tuning and early stopping. Furthermore, to assess the model’s robustness and generalizability, we apply 5-fold cross-validation on the training set. A detailed protocol is discussed in Section 4.3. To address class imbalance in the dataset, we use class-weighted cross-entropy loss and the Adam optimizer for training. We report metrics including accuracy (ACC), macro-F1 (MF1), and Cohen’s kappa (κ) averaged across folds. We designed the model input using three feature extraction methods: Modulation Spectrogram, Short-Time Fourier Transform (STFT), and Continuous Wavelet Transform (CWT). We used a single EEG channel (C4–M1), sampled at 100 Hz, for classification. We normalized all sleep stage labels as [W, N1, N2, N3, R] and discarded epochs marked P (Preparation stage). To ensure reproducibility, Table 2 details the specific parameters used for data preprocessing, feature extraction, and model training. All experiments were performed on a workstation equipped with an NVIDIA Quadro RTX 4000 GPU (NVIDIA Corporation, Santa Clara, CA, USA) and an Intel Xeon Silver 4214 processor (Intel Corporation, Santa Clara, CA, USA). The proposed model was implemented using Python 3.9 (Python Software Foundation, Beaverton, OR, USA) and the PyTorch 2.1.0 framework (Meta Platforms, Inc., Menlo Park, CA, USA). We utilized CUDA 11.8 (NVIDIA Corporation, Santa Clara, CA, USA) for GPU acceleration.

### 4.2. Deep Learning Models

We employ the EEGSNET model [39] in this study to train the spectrograms. This hybrid architecture: (i) uses residual CNN blocks to extract discriminative spectrotemporal features from EEG spectrograms that are effective for sleep stage classification, capturing different frequency patterns such as spindle activities in the N2 stage and high delta power in N3, then (ii) incorporates bidirectional LSTM to capture inter-stage transitions that characterize sleep architecture throughout the night. For additional supervision, we add an auxiliary classifier that guides the CNN to learn discriminative features before temporal modeling.

#### 4.2.1. Convolutional Neural Networks (CNNs)

CNNs are feedforward neural networks that differ from other deep neural networks due to their convolutional kernel structure, which enables them to extract local features by connecting input and output, making them effective for image-based models [43]. Based on this principle, we construct a feature-extraction module comprising four convolutional blocks. To reduce spatial resolution, each block contains convolutional layers, batch normalization layers, GELU activation, and Dropout2D with strided convolutions in deeper layers. This enables the network to learn hierarchical spectrotemporal patterns from input images. We include residual connections at each stage to stabilize training and retain low-level information. Finally, a global average pooling layer produces fixed-length feature embeddings for the sequence-learning module.

#### 4.2.2. Sequence Learning

While EEG provides direct insights into brain activity associated with sleep stages, accurate classification requires temporal continuity within successive epochs. To achieve this, we use CNNs to extract local features and employ LSTM to capture temporal dependencies across epochs. This hybrid design effectively combines spatial representation with sequence learning to improve sleep stage prediction.

#### 4.2.3. Bidirectional LSTM (BiLSTM)

Recurrent neural networks (RNNs) are designed for sequence learning but suffer from vanishing gradients, which limit model efficiency [44]. Long short-term memory networks (LSTMs) address this issue by incorporating gated mechanisms to regulate information flow and retain relevant long-term dependencies [45]. Bidirectional LSTMs (BiLSTMs) learn temporal dependencies in both forward and backward directions, making them particularly efficient for sleep staging tasks where inter-stage transitions depend on surrounding context [46]. In this work, we feed the neural network’s feature vectors into a two-layer BiLSTM at each epoch. Each BiLSTM layer consists of 128 hidden units in both directions, yielding a 256-dimensional representation per epoch. For a given subject recording, we construct a sequence $S_{t}$ of T=10 consecutive spectrograms. These sequences are extracted using a stride of S=5 epochs. There is a 50% overlap between consecutive input sequences due to the sliding window approach with S<(T×30s). This overlapping strategy serves as an implicit data augmentation technique, enabling the model to learn temporal dependencies across different positions within a sequence window. We employ a many-to-many architecture, where for each epoch in sequence, BiLSTM outputs a prediction. The 256-dimensional output at each step is passed through a shared linear layer to predict the respective sleep stage. This architecture allows the network to capture the inter-stage transition dependencies and characterize the progression of sleep stages over time, while maximizing the utility of every labeled epoch in the sequence.

#### 4.2.4. Auxiliary Classifier

Attention mechanisms highlight the most relevant features in sequential data while reducing the influence of less important information [47]. In this model, an auxiliary classifier at the per-epoch level acts as an attention-like mechanism, ensuring that CNN features remain discriminative before BiLSTM processing.

### 4.3. Training Protocol

**Figure 3:**
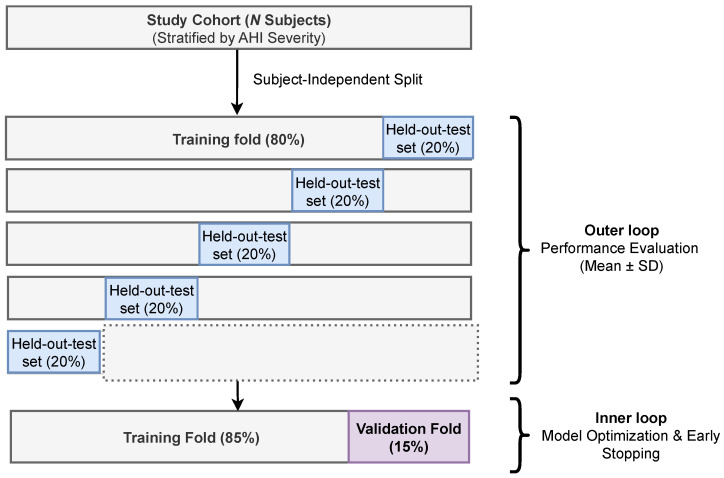
Schematic of the subject-independent 5-fold cross-validation protocol with nested internal validation for model optimization and early stopping.

To preserve the statistical robustness and prevent the subject-level data leakage, we adopted a Subject-Independent 5-fold stratified cross-validation scheme. To ensure that all the epochs from a specific subject appear exclusively either in the training, validation, or testing set for the given fold, the dataset was partitioned by subject ID. For each iteration of cross-validation, the data partitioning was organized as follows:

- Testing Set (20%): For final performance evaluation, a held-out subset was reserved exclusively to report the that can be seen in Section 5.
- Training and Validation Set (80%): The remaining subjects were utilized for model optimization. From this subset, we applied a stratified shuffle-split to allocate 15% of subjects to an internal Validation Set for hyperparameter tuning, with the remaining 85% used for training.
- Model Selection: We implemented an early-stopping mechanism based on the validation macro-F1 score to mitigate overfitting. The model was evaluated after every three epochs on the internal Validation Set, and training was halted if no improvement was observed for 10 consecutive evaluations (patience). The model state achieved the highest validation macro-F1, which was then restored and used for final inference on the held-out test set. This protocol was repeated for all 5-folds, with final results reported as mean ± standard deviation. This training workflow is illustrated in Figure 3.

### 4.4. Evaluation Metrics

We assess overall model performance using three evaluation metrics: Accuracy (ACC), Cohen’s kappa coefficient κ, and macro-average F1 score (MF1).

1. Accuracy (ACC): Accuracy measures the proportion of correctly classified epochs across all sleep stages. However, in imbalanced datasets such as sleep data, this metric can be misleading as it tends to bias toward the majority class and gives an inflated impression of performance [48]. While accuracy provides a general overview of model performance, it fails to capture how well the model performs on minority classes, which is critical for comprehensive sleep stage assessment.
2. Macro-average F1 Score (MF1): To address the limitations of accuracy, we use the F1-score, which balances precision and recall. The macro F1-score assigns equal weights to all classes, offering a more balanced evaluation of model performance, particularly for minority stages such as N1 and N3. We define the F1-score for class *i* as:
  $$F1_{i}=\frac{2\times ({\mathrm{Precision}}_{i}\times {\mathrm{Recall}}_{i})}{{\mathrm{Precision}}_{i}+{\mathrm{Recall}}_{i}},$$
  and compute the macro F1 as:
  $$MF1=\frac{1}{l}\sum_{i=1}^{l}F1_{i},$$
  where *l* is the number of sleep stages. This metric ensures that model performance on underrepresented classes receives equal consideration alongside that of majority classes.
3. Cohen’s Kappa Coefficient (κ): We use Cohen’s kappa coefficient to evaluate the level of agreement between predicted and actual sleep stages after adjusting for chance agreement [49]. In the context of sleep staging, evaluating kappa is crucial as it assesses how consistently the model reproduces stage boundaries and transitions relative to expert annotations. Unlike accuracy, kappa accounts for chance agreement, providing a more robust measure of classification performance. Higher kappa values indicate stronger agreement between model predictions and ground-truth labels, reflecting the model’s ability to capture the complex temporal patterns of sleep architecture.

### 4.5. Benchmark Systems

To evaluate the effectiveness of modulation spectrograms for sleep stage classification, we establish comprehensive baseline methods that represent current standard practices in the field. Our benchmark systems serve two critical purposes: (1) gauging the performance improvements achieved by the proposed framework, and (2) testing the complementarity of modulation features to existing feature representations. We implement two established time–frequency feature extraction methods as baselines:

1. Short-Time Fourier Transform (STFT): For the STFT baseline, we utilized a 30-s window length. This configuration strictly follows the standard preprocessing pipeline established in previous benchmarks [39], ensuring our baseline represents the current state-of-the-art. We explicitly maintained this 30-s window rather than extending it to 60 s because the STFT relies on signal stationarity. Extending the window would introduce non-stationarity, thereby compromising spectral resolution and reducing classification accuracy. Furthermore, following the benchmark [39], we resampled the EEG channel C4–M1 to 64 Hz and set the maximum analysis frequency to 32 Hz to capture EEG oscillations influencing sleep stage transitions. We compute spectrograms using a Hamming window with overlapping segments, apply power scaling, and restrict the frequency range to 0–32 Hz. Lastly, resultant images are cropped to 76×60×3 to remove text and redundant blank areas.
2. Continuous Wavelet Transform (CWT): To provide a robust comparison against established time–frequency representations, we implemented a CWT baseline. To be consistent with STFT baseline and AASM clinical scoring standard, we employed a 30-s window length [30,50]. This alignment with the standard duration ensures the CWT baseline strictly adheres to established protocols, thereby preventing methodological discrepancies introduced by arbitrary window sizes. Preprocessing was matched to the STFT pipeline: we resampled channel C4–M1 to 64 Hz, restricted the maximum frequency to 32 Hz, and normalized each 30-s epoch. We then transform the signal using an analytic Morlet (’amor’) filterbank with 12 frequency divisions per octave across the 0–32 Hz range, providing multi-resolution time–frequency representations. The resulting scalogram is normalized, and low-power scaling is applied, and the image is then resized to 76×60×3 for input to the neural network.

## 5. Results and Discussion

In this section, we present experimental results for sleep stage classification across AHI-stratified populations. We first conduct ablation studies to identify optimal modulation-spectrogram configurations as discussed in Section 5.1. Next, we compare CNN-only versus CNN + biLSTM architectures in Section 5.2 to evaluate the necessity of temporal modeling. Finally, we compare modulation spectrograms against established baseline methods (STFT and CWT) across all AHI cohorts in Section 5.3. In the following tables, bold typeface indicates the best performance metrics achieved among the compared methods.

### 5.1. Ablation Study: Optimal Modulation Spectrogram Configuration

To maximize the effectiveness of modulation spectrograms for sleep staging, we systematically investigate two critical design parameters: input image resolution and temporal window length. These parameters directly impact the representation’s ability to capture fine-grained carrier-modulation patterns and slow temporal dynamics characteristic of sleep phenomena. We conduct all ablation studies on the Normal AHI cohort to establish optimal configurations before evaluating across clinical severity groups.

Impact of adaptive window on data retention: The key advantage of the proposed adaptive windowing method (chosen as the optimal temporal configuration) ensures complete data retention. In the conventional fixed-window approaches, epochs are often discarded or misclassified when they fail to meet stationarity criteria, particularly during sleep transitions, where inter-rater reliability is historically low [15,51]. Previous methodologies enforcing strict label consistency over extended windows would necessitate discarding these transitional epochs.

By switching to the 30-s fallback window during transitions, our pipeline retained 100% of the original 30-s scoring epochs. In our dataset, the distribution of window utilization was approximately 83.3% for the Extended Window (66,647 epochs, representing stable sleep periods) and 16.7% for the Fallback Window (13,317 epochs, representing transition boundaries). This ensures that stage transitions—often the most challenging segments to score automatically—are strictly preserved rather than filtered out.

#### 5.1.1. Effect of Image Resolution

Modulation spectrograms encode information across two dimensions: carrier frequency (spectral content, 0.5–40 Hz) and modulation frequency (amplitude variation rate, 0–10 Hz). Higher image resolutions preserve finer details in both dimensions but increase computational cost and may introduce unnecessary complexity. We evaluate three resolutions using 60-s windows on the Normal AHI cohort:

- 76 × 60 × 3: Matches baseline STFT/CWT dimensions for direct comparison
- 152 × 120 × 3: Doubles resolution to preserve finer carrier-modulation patterns
- 224 × 224 × 3: Standard CNN input size, enabling potential transfer learning

Table 3 presents the results. As image size increases from 76 × 60 to 152 × 120, we observe substantial improvements in accuracy from 65.6% to 73.9%, Cohen’s kappa from 52.6% to 62.4%, and macro-F1 from 55.2% to 64.3%. This improvement suggests that the baseline resolution compresses critical modulation information, particularly slow amplitude variations that characterize sleep spindles and K-complexes. The 152 × 120 resolution adequately captures these patterns without introducing noise or excessive computational burden. However, further increasing resolution to 224 × 224 does not provide any additional gains and, in some cases, shows slight performance degradation in Cohen’s kappa from 62.4% to 61.1%. This suggests that relevant carrier-modulation information for sleep staging is adequately represented at 152 × 120, and higher resolutions may introduce unnecessary complexity that hinders generalization without adding discriminative information.

#### 5.1.2. Effect of Window Length

Modulation analysis captures slow variations in spectral energy patterns that become more apparent with longer observation windows [12]. Sleep phenomena such as spindle bursts, K-complex sequences, and slow-wave patterns often exhibit temporal structures spanning multiple 30-s epochs. We evaluate three temporal window configurations using the optimal 152 × 120 × 3 resolution:

- 30-s window: Single epoch (matches baseline methods).
- 60-s window: Formed by merging two consecutive epochs (t,t+1) only if they share the identical sleep stage label.
- 90-s window: Formed by merging three consecutive epochs (t−1,t,t+1) only if all three share the identical sleep stage label.

Table 4 demonstrates that the standard 30-s window is insufficient to capture the complete dynamics of amplitude modulation patterns essential for sleep stage discrimination. Results show a substantial performance drop, with a macro F1 of 55%, which fails to outperform the STFT baseline. This empirically confirms that an extended 60-s integration time is inherent to the method: it provides the necessary temporal context to resolve slow (<1 Hz) physiological rhythms—such as the 0.5–2 Hz modulation of spindles and slow-wave activity—that shorter epochs truncate. Thus, the proposed ‘60 s Modulation’ framework functions as an indivisible processing unit, where the extended duration is essential to satisfy the resolution constraints of the representation. Furthermore, 90-s windows show almost the same performance in terms of accuracy and occasionally degrade macro-F1 performance when we compare it with 60-s windows. Excessively long windows introduce two problems: (1) increased likelihood of including epochs with different sleep stage labels, introducing label noise that confuses the classifier, and (2) averaging effects that blur stage transition boundaries, reducing temporal specificity. The 60-s window provides an optimal balance between capturing relevant modulation dynamics and maintaining label consistency.

### 5.2. Deep Learning Based Model Comparison

To demonstrate the necessity of temporal sequence modeling for sleep stage classification, we compare a CNN architecture with the hybrid CNN + BiLSTM approach, termed EEGSNet. While CNNs effectively extract spatial patterns from spectrograms, sleep staging fundamentally requires modeling temporal dependencies across consecutive epochs, which captures the sequential nature of sleep architecture transitions throughout the night.

Table 5 presents results for both architectures across all three feature extraction methods on the Normal AHI cohort. The CNN-only baseline was simplified by removing BiLSTM and attention modules from EEGSNet to isolate the contribution of temporal modeling. Results demonstrate that EEGSNet consistently and substantially outperforms the CNN-only baseline across all feature types, as shown in Table 5. Sleep staging requires understanding not only what patterns are present in individual epochs but also how these patterns evolve across time information that BiLSTMs explicitly model through their recurrent connections. The auxiliary classifier in EEGSNet further ensures that CNN features remain discriminative before temporal aggregation, providing an attention-like mechanism that guides feature learning. This emphasizes that temporal modeling is not optional but essential for robust sleep staging, regardless of feature quality.

### 5.3. Comparison of Proposed Method with BASELINE

Here, we present the classification results achieved with the proposed and benchmark features on the DreamT dataset for sleep stage classification task. We conducted all experiments using EEGSNet [39] as the backbone architecture to ensure fair comparison across feature extraction methods. We implemented STFT and CWT as baseline methods following the original preprocessing pipeline [39]. Furthermore, evaluating performance across AHI-stratified cohorts addresses a critical gap in sleep staging research. All methods utilized identical subject-independent training and evaluation procedures as described in Section 4. We employed 5-fold cross-validation with no subject overlap between folds. Training used the Adam optimizer with class-weighted cross-entropy loss to address class imbalance, and employed identical early stopping criteria and hyperparameter settings across all feature types.

**Figure 4:**
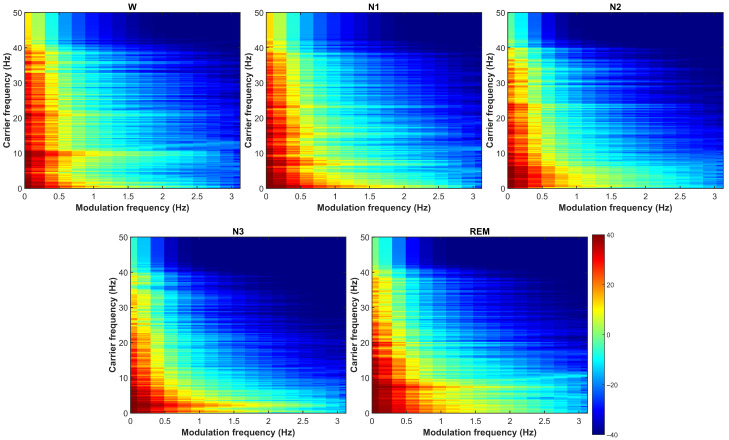
Modulation spectrograms of the EEG signal (C4–M1) for the Normal AHI group, illustrated using data from participant S011 across the five sleep stages: W, N1, N2, N3, and REM. A shared power scale (dB) appears next to the REM panel.

Figure 4 illustrates modulation spectrograms extracted from the C4–M1 EEG channel across all five sleep stages from a participant in the Normal AHI group. These visualizations reveal that modulation representations capture slow-varying modulation patterns (0–1.6 Hz) evolving along the carrier frequency axis (0–50 Hz). It is crucial to note that resolving these low-frequency rates (<1 Hz) inherently requires the 60-s integration window used in our framework. Applying such extended windows to baselines would, conversely, introduce significant non-stationarity and spectral blurring. Therefore, the performance gains reported herein reflect a necessary coupling between the rate-based representation and extended temporal context, rather than being attributable solely to the feature extraction mathematics in isolation. Distinct spectrotemporal patterns clearly separating different sleep stages, such as Wake and N1, demonstrate stronger high-frequency carrier energy (>15 Hz) and broader modulation activity, reflecting the mixed-frequency patterns and alpha activity characteristic of relaxed wakefulness and light sleep transitions. N2 exhibits enhanced modulation specifically around spindle-related carrier bands (10–16 Hz), capturing the characteristic waxing-waning amplitude envelopes of sleep spindles that define this stage. N3 is dominated by low-frequency carriers (<4 Hz) with minimal modulation content, reflecting the stable, high-amplitude slow-wave activity that distinguishes deep sleep. REM shows a mixed pattern with elevated higher-frequency carrier energy but weak low-modulation content, consistent with the desynchronized, low-amplitude brain activity characteristic of REM sleep. These variations in intensity and distribution across sleep stages indicate that modulation representations preserve physiologically meaningful distinctions that should facilitate automated classification.

We performed comprehensive experiments across all four AHI-stratified subgroups: Normal, Mild, Moderate, and Severe, as shown in Table 6, Table 7, Table 8 and Table 9.

1. Normal AHI Group. Table 6 presents results for participants without clinically significant sleep apnea. Across the five-fold cross-validation, the proposed framework demonstrated robust performance, achieving a mean accuracy of 0.74 ± 0.08. In terms of agreement and class-wise balance, the model yielded a Cohen’s kappa of 0.62 ± 0.11 and a Macro-F1 score of 0.63 ± 0.06, confirming that the method generalizes well across different data partitions.
2. Mild AHI Group. Table 7 reveals that performance advantages amplify in the Mild AHI group. The proposed framework achieved substantial gains, outperforming the STFT baseline by 7% in accuracy and 12% in Macro-F1. Furthermore, it surpassed the CWT method by 5% in accuracy, coupled with a 12% macro-F1. The proposed framework improvement over baselines suggests that modulation features capture patterns that are more resilient to the increased arousals and stage fragmentation present in mild OSA. Despite sleep irregularities characteristic of this clinical population, modulation-based feature extraction remained robust and provided superior discrimination of sleep stage transitions.
3. Moderate AHI Group. Results for the Moderate AHI group, shown in Table 8, reveal a slightly different pattern where modulation spectrograms performed comparably to baselines rather than being superior. While baselines achieved marginally higher accuracy, modulation maintained competitive macro-F1, indicating more balanced per-stage performance despite lower overall accuracy. This reduced performance advantage likely reflects the increased sleep variability and fragmentation in moderate OSA, which may limit the effectiveness of detailed modulation pattern analysis. Notably, CWT’s higher accuracy, coupled with equal macro-F1 and higher standard deviation, suggests a dependency on majority classes (particularly N2 and Wake), whereas modulation spectrograms maintained greater class-level balance. This trade-off between overall accuracy and balanced class performance represents an important consideration for clinical applications, where reliable detection of all sleep stages, not just dominant ones, is essential for comprehensive sleep architecture assessment.
4. Severe AHI Group. Table 9 demonstrates a comparison of the proposed and benchmark approaches in the Severe AHI group. Modulation spectrograms achieved 0.66 ± 0.05 accuracy, 0.52 ± 0.05 Cohen’s kappa, and 0.57 ± 0.05 macro-F1, substantially outperforming STFT and CWT. Both baseline methods showed marked performance degradation in this challenging population, with STFT experiencing particularly sharp drops in Cohen’s kappa (a 15 percentage-point decrease from Normal to Severe) and macro-F1 (a 11-point decrease). These drops indicate baseline methods’ sensitivity to sleep fragmentation and inability to handle severe irregularities characteristic of advanced OSA. In contrast, modulation-based representations showed a more gradual decline in performance (7.8 percentage-point decrease in accuracy, 6-point decrease in macro-F1 from Normal to Severe), illustrating greater resilience to increased signal noise and irregular sleep patterns commonly observed in severe OSA patients.

Examining performance trends across the complete AHI spectrum reveals important patterns about feature robustness and clinical applicability. Across the Normal, Mild, and Severe AHI groups, modulation spectrograms consistently outperformed both baselines across all metrics, demonstrating robustness to altered sleep architecture in clinical populations. While all methods experienced performance decline as sleep variability increased with apnea severity, a natural consequence of increasingly disrupted sleep architecture, the rate of decline differed markedly between feature types. The reduction in macro-F1 for modulation features was noticeably more gradual (6 percentage points from Normal to Severe) than for baseline methods (STFT: 11 points, CWT: 17 points). This resilience indicates that the modulation-based framework remains more stable despite progressive sleep fragmentation associated with higher AHI levels. Furthermore, the improved macro-F1 scores for modulation features better reflect successful recognition of difficult-to-classify minority classes, particularly N1 and N3, which are clinically crucial for comprehensive sleep assessment yet often underperform in automated systems. The overall performance gap between Normal and Severe AHI groups across all methods is approximately 6–10% lower accuracy in the Severe group, highlighting the fundamental challenge of automated sleep staging in participants with severe obstructive sleep apnea. This finding underscores the critical importance of clinical validation with stratified populations.

#### 5.3.1. Per-Stage Performance Analysis

**Figure 5:**
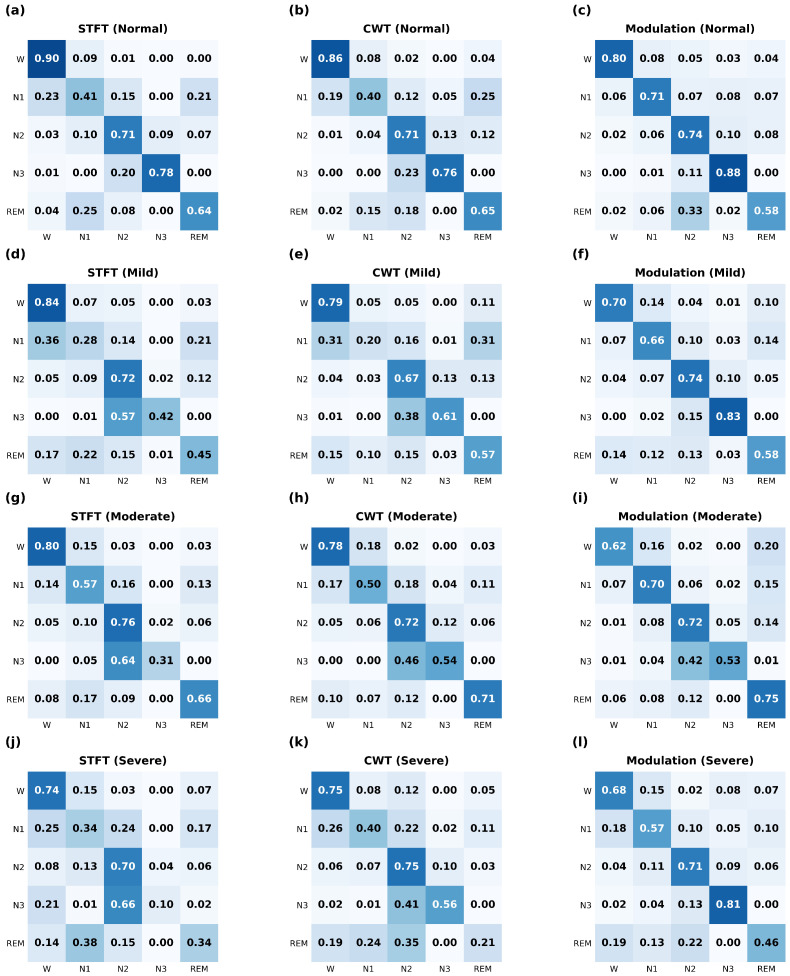
Confusion matrices showing sleep stage classification performance across Normal, Mild, Moderate, and Severe AHI groups using three feature extraction methods: STFT, CWT, and Modulation. Rows represent AHI groups, and columns represent feature methods. The color intensity represents the normalized confusion matrix values, where darker blue indicates a higher proportion of classification agreement (closer to 1.0) and lighter blue indicates lower agreement (closer to 0).

To understand where modulation spectrograms provide specific advantages over baseline methods, we analyzed per-stage classification performance using confusion matrices. Figure 5 presents confusion matrices for all twelve experimental conditions: three feature extraction methods, STFT, CWT, and modulation spectrograms, evaluated across four AHI severity groups.These matrices reveal nuanced patterns in how different feature representations perform across sleep stages and clinical populations. Across the Normal, Mild, and Severe AHI groups, modulation-based features consistently outperformed STFT and CWT representations, while in the Moderate group, performance was comparable to baselines.

As shown in Figure 5, a particularly noteworthy finding is the substantial improvement in N1 classification achieved with modulation spectrograms. Across all AHI groups, N1 F1-scores were consistently and markedly higher: 0.71 in the Normal group, 0.66 in the Mild group, 0.70 in the Moderate group, and 0.57 in the Severe group, substantially better than both CWT and STFT performance. The superior N1 performance suggests that modulation spectrograms, through their explicit decomposition into carrier and modulation frequencies via a modulation filterbank, more effectively capture the slow-varying amplitude patterns and subtle transitional dynamics that characterize this stage. While STFT and CWT detect the instantaneous frequency content during N1 epochs, they do not explicitly model how amplitude varies over time, a factor that appears particularly discriminative for this transitional stage.

**Figure 6:**
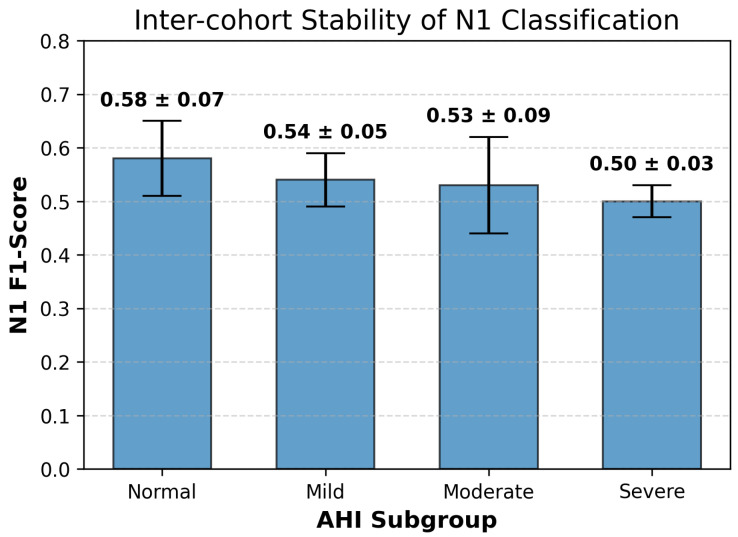
Mean F1-scores (±SD) for the N1 sleep stage across the four AHI-based clinical subgroups.

Furthermore, we assessed whether the improved sensitivity to N1 compromised the classification accuracy of other sleep stages. N1 vs. N2/N3: The results indicate that N1 gains were not zero-sum. While N1 sensitivity improved by +23% in the severe cohort, N2 performance remained stable (70%→71%). Notably, the proposed framework drastically improved N3 (Deep Sleep) identification (10%→81%), correcting the baseline’s tendency to conflate fragmented N3 with N2. Wake Specificity: A trade-off was observed regarding Wake sensitivity, which decreased in the severe group (74%→68%). However, this aligns with the clinical objective of reducing false-negative sleep results. The confusion matrix confirms that the proposed model reduced the False Wake rate (N1 misclassified as Wake) from 25% to 18%, suggesting more rigorous detection of sleep onset boundaries. To verify that the improved performance on the N1 sleep stage was not driven by a limited subset of subjects or specific data partitions, we analyzed the consistency of N1-specific F1-scores across the five cross-validation folds within each stratified cohort. As illustrated in Figure 6, the proposed method demonstrated high stability across the severity spectrum. The model achieved a mean N1 F1-score of (0.58 ± 0.07) in the Normal group and maintained robust performance in the Mild (0.54 ± 0.05), Moderate (0.53 ± 0.09), and Severe (0.50 ± 0.03) cohorts. It is worth noting that low standard deviations—particularly in the Severe group (SD = 0.03)—indicate that the model’s ability to detect N1 transitions is consistent across random subject partitions and is not reliant on outliers or “easy” cases, confirming the generalized efficacy of the modulation-based feature extraction for detecting sleep onset and fragmentation.

In the Severe AHI group, the limitations of conventional time–frequency methods became particularly pronounced, with both the STFT and the CWT exhibiting substantial confusion patterns that illuminate their failure modes. STFT exhibited marked confusion in sleep stage classification, frequently misclassifying N1 as Wake (25% confusion rate), N2 (24%), or REM (17%), and often misidentifying N3 as N2 (66%) or Wake (21%). This degradation stems from sleep fragmentation, a common feature of severe sleep abnormalities. The fragmentation of slow waves blurs the stable, high-amplitude, low-frequency patterns characteristic of N3, reducing the STFT’s discriminative capacity, since it fundamentally relies on detecting sharp, stable spectral features at specific frequencies and times.

Similarly, CWT struggled with classification in the Severe group, particularly for N1 (frequently confused with Wake 26%, N2 22%, or REM 11%) and REM (confused with N2 35%, N1 24%, or Wake 19%). While CWT’s multi-resolution analysis theoretically provides advantages for analyzing non-stationary signals, this benefit diminishes in severely fragmented sleep, where brief arousals and micro-transitions create highly irregular signal patterns that wavelet decompositions cannot consistently represent. The increased instability of transitional stages in patients with sleep disorders characterized by rapid, unpredictable stage switches and mixed physiological states creates signal characteristics that challenge the assumptions underlying wavelet-based time–frequency analysis. Interestingly, modulation-based methods remained comparatively stable under these challenging conditions, maintaining more precise stage boundaries even in the Severe group. This stability suggests that the modulation domain captures deeper amplitude-modulation structures that remain informative even in severely fragmented EEG. Rather than relying on stable frequency content at specific times (STFT’s approach) or consistent wavelet decomposition patterns (CWT’s approach), modulation analysis quantifies how amplitude varies over time, a property that appears more robust to the disruptions caused by severe sleep-disordered breathing.

The consistent performance differences observed across all methods between the Normal, Mild, Moderate, and Severe AHI groups provide compelling evidence for the critical importance of stratifying participants by clinical severity when developing and evaluating sleep staging algorithms. This finding addresses a fundamental gap in the sleep staging literature: most algorithms are validated primarily in healthy populations, despite clinical deployment predominantly in patient populations with sleep disorders.

#### 5.3.2. Clinical Implications

This improvement carries significant clinical implications because N1 represents a transitional stage between wakefulness and sleep, characterized by subtle, low-amplitude mixed-frequency patterns and brushing a bidirectional LSTM to incorporate future context for both human scorers and automated classification systems. While our framework demonstrated improvement in all four AHI subgroups, we emphasize performance in the severe apnea cohort as an essential benchmark for clinical utility, given the extreme sleep fragmentation observed in this group. In this challenging apnea-based group, the proposed modulation framework achieved N1 sensitivity of 57%, significantly outperforming STFT (34%) and CWT (40%) baselines. This improvement directly enhances the reliability of key clinical biomarkers, importantly, Sleep Onset Latency (SOL). In clinical practices, the transition from wake to N1 defines sleep onset; by accurately resolving this boundary, where conventional systems often fail due to spectral similarity, the proposed framework mitigates the systematic inflation of sleep onset latency estimates in insomnia and narcolepsy.

In sleep apnea, where recurrent apneic events are followed by micro-arousals, the model’s incorporation of a 60-s context facilitates identification of transient N1 transitions overlooked by traditional approaches, resulting in a finer-grained fragmentation index that better captures true sleep disruption. Accordingly, the framework is designed for offline automated scoring to assist post-hoc PSG analysis. Although feature computation is lightweight, the use of a bidirectional LSTM to incorporate future context (t+1…t+N) is a non-causal choice that prioritizes diagnostic accuracy and prediction stability over real-time latency.

Several limitations of this study merit acknowledgment and suggest directions for future research. First, following the baseline [39], we used only a single-channel EEG (C4–M1). This design choice establishes a performance lower bound suitable for resource-constrained environments such as Home Sleep Testing (HST). However, we acknowledge that in full clinical settings, multi-channel recordings could provide complementary spatial information to improve accuracy, particularly during ambiguous stages like N1. Second, while our ablation studies systematically evaluated image resolution and window length, many other hyperparameters, including subband definitions, modulation frequency ranges, and normalization strategies, remain unexplored. Third, the DREAMT dataset, though valuable for including well-characterized clinical populations with diverse AHI levels, comprises only 100 participants from a single sleep laboratory, limiting generalizability assessments across diverse age groups, comorbidities, and geographic regions. Future work should investigate extending the framework to multi-channel EEG montages (including F4 and O2 leads) and complementary polygraphic signals (EOG, EMG), comprehensive hyperparameter optimization, and validation on larger, multi-center datasets to strengthen these findings.

## 6. Conclusions

In this paper, we explored modulation spectrograms for automated sleep stage classification in the presence of confounding clinical factors, such as obstructive sleep apnea severity and sleep fragmentation. We systematically demonstrated how clinical severity degrades the performance of conventional time–frequency representations. To address this, we proposed a modulation-based feature representation with optimal configurations (60-s windows, 152 × 120 × 3 resolution) that significantly outperforms STFT and CWT baselines across most AHI-stratified cohorts. Finally, we showed that the proposed framework not only achieves higher accuracy but also superior robustness (lower variance) in severe clinical populations, highlighting the potential of modulation-based approaches for reliable and robust clinical sleep staging.

## Figures and Tables

**Table 1:** Subject distribution, class-wise epoch counts, and stratified data split (Train/Validation/Test).

Dataset	Subjects (Train/Validation/Test)	W	N1	N2	N3	REM	Total
DREAMT	100	20,041	8818	39,953	2704	8387	79,903
Normal (<5 events)	26 (18/4/4)	5638	1715	10,246	567	2508	20,674
Mild (5–14)	25 (17/4/4)	4809	1971	10,295	906	2294	20,275
Moderate (15–29)	24 (16/4/4)	4711	2189	9816	647	1907	19,270
Severe (≥30)	25 (17/4/4)	4883	2943	9596	584	1678	19,684

**Table 2:** Detailed configuration of preprocessing, feature extraction, and model training parameters for all compared baselines.

Parameter	STFT (Baseline)	CWT (Baseline)	Modulation (Proposed)
1. Preprocessing & Input			
Sampling Rate	100 Hz		
Window Duration	30 s (Fixed)	30 s (Fixed)	Adaptive (30 s/60 s)
Stride	30 s	30 s	30 s
2. Feature Extraction			
Spectral Resolution	256 freq bins	64 scales	44 freq bins
Window Function	Hamming	Morlet	Rectangular
Frequency Range	0–32 Hz	0–32 Hz	0.5–30 Hz
3. Model Training			
Architecture	CNN + BiLSTM	CNN + BiLSTM	CNN + BiLSTM
Sequence Length	T=10	T=10	T=10
Optimizer	Adam ($\alpha =1\times 10^{-3}$, $\beta_{1}=0.9,\beta_{2}=0.999$)		
Weight Decay	$1\times 10^{-4}$		
Stopping Criterion	Patience = 10 (Metric: Val Macro-F1)		

**Table 3:** Effect of image resolution on modulation spectrogram performance (Normal AHI group, 60-s window).

Image Size	Accuracy	Kappa	Macro-F1
76 × 60 × 3	0.656	0.526	0.552
152 × 120 × 3	**0.739**	**0.624**	**0.643**
224 × 224 × 3	0.724	0.611	0.628

**Table 4:** Effect of window length on modulation spectrogram performance (Normal AHI group, 152 × 120 × 3 resolution).

Window Length	Accuracy	Kappa	Macro-F1
30-s	0.669	0.526	0.550
**60-s**	**0.739**	**0.624**	**0.643**
90-s	0.731	0.610	0.635

**Table 5:** Comparison of CNN-only baseline versus EEGSNet (CNN + BiLSTM) architecture.

Method	Model	Window	Resolution	Acc	Kappa	MF1
STFT	CNN	30 s	76 × 60 × 3	0.676	0.498	0.514
STFT	EEGSNet	30 s	76 × 60 × 3	**0.728**	**0.614**	**0.610**
CWT	CNN	30 s	76 × 60 × 3	0.629	0.459	0.490
CWT	EEGSNet	30 s	76 × 60 × 3	**0.718**	**0.599**	**0.591**
Modulation	CNN	60 s	152 × 120 × 3	0.659	0.512	0.560
Modulation	EEGSNet	60 s	152 × 120 × 3	**0.739**	**0.625**	**0.643**

**Table 6:** Comparison of proposed and baseline approaches for Normal AHI Group.

Method	Window	Image Size	Accuracy (Mean ± SD)	Kappa (Mean ± SD)	Macro-F1 (Mean ± SD)
STFT	30 s	76 × 60 × 3	0.72 ± 0.11	0.61 ± 0.14	0.59 ± 0.11
CWT	30 s	76 × 60 × 3	0.73 ± 0.04	0.61 ± 0.05	0.59 ± 0.07
**Modulation**	**60 s**	**152 × 120 × 3**	**0.74 ± 0.08**	**0.62 ± 0.11**	**0.63 ± 0.06**

**Table 7:** Comparison of proposed and baseline approaches for Mild AHI Group.

Method	Window	Image Size	Accuracy (Mean ± SD)	Kappa (Mean ± SD)	Macro-F1 (Mean ± SD)
STFT	30 s	76 × 60 × 3	0.64 ± 0.09	0.49 ± 0.10	0.51 ± 0.08
CWT	30 s	76 × 60 × 3	0.66 ± 0.11	0.51 ± 0.13	0.51 ± 0.12
**Modulation**	**60 s**	**152 × 120 × 3**	**0.71 ± 0.05**	**0.59 ± 0.06**	**0.63 ± 0.06**

**Table 8:** Comparison of proposed and baseline approaches for Moderate AHI Group.

Method	Window	Image Size	Accuracy (Mean ± SD)	Kappa (Mean ± SD)	Macro-F1 (Mean ± SD)
STFT	30 s	76 × 60 × 3	0.70 ± 0.06	0.56 ± 0.08	0.59 ± 0.06
CWT	30 s	76 × 60 × 3	0.72 ± 0.05	0.59 ± 0.07	0.59 ± 0.09
**Modulation**	**60 s**	**152 × 120 × 3**	**0.68 ± 0.07**	**0.56 ± 0.09**	**0.59 ± 0.06**

**Table 9:** Comparison of proposed and baseline approaches for Severe AHI Group.

Method	Window	Image Size	Accuracy (Mean ± SD)	Kappa (Mean ± SD)	Macro-F1 (Mean ± SD)
STFT	30 s	76 × 60 × 3	0.65 ± 0.05	0.46 ± 0.09	0.48 ± 0.09
CWT	30 s	76 × 60 × 3	0.62 ± 0.08	0.44 ± 0.10	0.42 ± 0.06
**Modulation**	**60 s**	**152 × 120 × 3**	**0.66 ± 0.05**	**0.52 ± 0.05**	**0.57 ± 0.05**

## Data Availability

The DREAMT dataset used in this study is available on PhysioNet under restricted access. The implementation code is publicly available at https://github.com/Unaiza4/modulation-based-sleep-classification (accessed on 20 November 2025). The processed spectrogram and modulation feature representations generated in this study are available from the corresponding author upon reasonable request.
